# Single-cell and transcriptome analyses revealed CTHRC1 a potential therapeutic target mediating invasion and tumor microenvironment in TNBC: experimental validation

**DOI:** 10.3389/fimmu.2025.1534981

**Published:** 2025-03-11

**Authors:** Hong Wan, Zichen Ling, Yuwei Xie, Han Jiang, Zhifan Ruan, Dashuai Yang, Xiaowei Yang, Jing Pei

**Affiliations:** ^1^ Department of General Surgery, The First Affiliated Hospital of Anhui Medical University, Hefei, China; ^2^ Department of Breast Surgery, The First Affiliated Hospital of Anhui Medical University, Hefei, China; ^3^ Department of Hepatobiliary Surgery, Renmin Hospital of Wuhan University, Wuhan, China

**Keywords:** TNBC, invasion, CAFs, tumor microenvironment, M2-type macrophages

## Abstract

**Background:**

Investigating the pivotal role of CTHRC1 in the tumor microenvironment of triple-negative breast cancer (TNBC).

**Method:**

The RNA transcriptomic data obtained from the Cancer Genome Atlas and single-cell sequencing data from TNBC in Gene Expression Omnibus (GEO) were acquired and subjected to analysis. A comprehensive investigation was conducted with a specific focus on characterizing CTHRC1 in TNBC and its correlation with invasive genes. Furthermore, additional analyses were performed to explore the relationship between CTHRC1, tumor immune cell infiltration, and immunotherapy in TNBC. The expression of CTHRC1 in the tumor microenvironment, cellular differentiation, and cellular communication was systematically analyzed using single-cell data from TNBC.

**Result:**

The expression of CTHRC1 in patients with TNBC gradually increases concomitantly with the progression of tumor T-stage and N-stage. Simultaneously, there is a concurrent increase in the expression of most invasive gene sets. Furthermore, there is a significant augmentation in both infiltration abundance and activity of M2-type macrophages associated with elevated levels of CTHRC1 expression. Single-cell data reveal an upregulated expression of the invasive gene set in CTHRC1-positive cancer associated fibroblasts (CAFs), thereby modulating their interaction with M2-type macrophages. Multiple immunofluorescence analyses confirmed that CTHRC1 modulates immune cell infiltration and tumor cell invasion through the mediation of CAFs.

**Conclusion:**

CTHRC1 was a molecule that exhibits characteristic expression in TNBC. CTHRC1 positive CAFs exert regulatory effects within the immunosuppressive microenvironment of TNBC by modulating M2-type macrophages.

## Background

Breast cancer ranks as the second leading cause of cancer-related fatalities and represents the most prevalent malignancy among women (1). Breast cancer can be categorized into three primary subtypes based on the expression levels of estrogen receptor (ER), progesterone receptor (PR), and human epidermal growth factor receptor 2 (HER2): hormone receptor-positive, HER2-positive, and triple-negative breast cancer (TNBC) (2). Patients diagnosed with triple-negative breast cancer exhibit a considerably elevated 5-year recurrence rate and demonstrate the most unfavorable prognosis, compared to the other two pathological subtypes (3). Triple-negative breast cancer constitutes approximately 15 percent of breast cancer cases, and its distinct molecular expression profile renders patients unresponsive to endocrine and targeted therapies (4). Surgery and chemotherapy are still the mainstream of treatments (5).

Single-cell and bulk transcriptomic data offer significant advantages in the field of oncology: 1) Enabling the analysis of gene expression at the single-cell level, thereby uncovering intercellular heterogeneity. 2) Facilitating precise identification and classification of diverse cell types and states, which aids in the discovery of novel cellular subpopulations. 3) Revealing spatial heterogeneity within tissues or tumors. 4) Providing a high degree of data standardization, which enhances cross-study comparisons and integrations.

The extracellular matrix (ECM) protein CTHRC1 engages in intricate interactions with diverse intracellular and extracellular matrices through distinct secretory mechanisms (6). CTHRC1 exhibits robust expression in various tumor types and facilitates cancer cell proliferation, invasion, and metastasis (7, 8). Additionally, it has been documented that CTHRC1 plays a role in the regulation of macrophage recruitment (9, 10).

This study investigates the distinct expression profile of CTHRC1 in TNBC, elucidating its association with invasion-related genes. Leveraging single-cell data from triple-negative breast cancer, we comprehensively explore the role of CTHRC1 within the tumor microenvironment, with a specific focus on immunoregulation and intercellular interactions.

## Method

This study incorporates transcriptomic data obtained from 33 tumor tissues, which were log2 transformed, downloaded from The Cancer Genome Atlas (TCGA). The Gene Expression Omnibus (GEO) database encompasses transcriptomic data derived from tumor tissues as well as single-cell sequencing data. Transcriptomic profiles of TNBC patients were retrieved from GSE58812, GSE164458, and GSE157284 (11–13). Additionally, the study incorporated single-cell transcriptome data obtained from 9 patients with TNBC through the GSE176078 dataset (14). CancerSea has acquired a compilation of 14 genes associated with the biological characteristics of tumors from http://biocc.hrbmu.edu.cn/CancerSEA/ (**Supplementary Table 1**).

### Pan-cancer analysis

We have generated transcriptome expression profiles for 33 tumor tissues, excluding those lacking information on paracancerous tissues. By analyzing the data from both tumor and paracancerous tissues, we initially compared the alterations in CTHRC1 expression levels. Additionally, we extracted the expression patterns of the invasion gene set across various tumor tissues. Subsequently, we conducted a correlation analysis between CTHRC1 expression in different tumors and the invasion gene set. Finally, we assessed the association between CTHRC1 expression in diverse tumors and gene expression of immunosuppressant therapeutic targets.

### Clinical characterization of CTHRC1 in TNBC

The distinct characteristics of CTHRC1 in TNBC and non-TNBC were comprehensively elucidated. Breast cancer samples were classified into TNBC and non-TNBC subtypes, followed by an initial analysis of the association between CTHRC1 expression and tumor T-stage. The expression of CTHRC1 was stratified into high and low groups based on the median value. We examined the differential expression of the invasive gene set in conjunction with the trend in CTHRC1 expression. Furthermore, we corroborated this trend by analyzing expression data from multiple GEO databases for patients with TNBC.

### Immune cell infiltration and immunotherapeutic features

Immunotherapy is a pivotal therapeutic modality for tumor patients, and thus we investigated the correlation between CTHRC1 expression and immune cell infiltration in TNBC patients (15). The abundance and activity of immune cell infiltration in triple-negative breasts were comprehensively analyzed using advanced algorithms including CIBERSORT, ssGSEA, and ESTIMATE. TIDE has emerged as a widely adopted tool for predicting immunotherapy response in tumor patients (16, 17). Moreover, we conducted predictive analyses to assess the potential efficacy of targeting CTHRC1 in patient immunotherapy.

### Single-cell data processing

The dataset GSE176078 consists of transcriptomic data obtained from single cells of nine patients diagnosed with TNBC. Initially, the data were processed individually to create Seurat objects. Rigorous quality control measures were implemented on individual samples to exclude those exhibiting (1) mitochondrial gene expression exceeding 10%, (2) bicellular contamination, and (3) erythrocyte gene expression surpassing 3%. Furthermore, cell cycle effects were accounted for and eliminated from the analysis. Subsequently, the harmony function was employed to integrate the datasets, followed by dimensionality reduction using Unified Mobility Approximation and Project (UMAP), enabling visualization of clustered cells in a two-dimensional map. Cell types were identified utilizing “SingleR” along with relevant high-quality literature references (18–21). Finally, specific cell types of interest underwent further dimensionality reduction clustering and comprehensive annotation at the subpopulation level.

### GSVA enrichment analysis

GSVA enrichment analysis is widely used to assess the activity scores of gene sets in samples. We evaluated the correlation between 14 gene sets related to cell biology and CTHRC1. Firstly, we selected the expression matrix of TNBC and used the GSVA algorithm to calculate the scores of the 14 gene sets in the samples. Finally, we analyzed the correlation between CTHRC1 and their activity levels. In single-cell data, we annotated detailed cell types present in the tumor microenvironment of triple-negative breast cancer. Initially, we extracted “RNA” data from single-cell sequencing results and then assessed the activity scores of these gene sets in different cellular subsets using GSVA method.

### Analysis of cell differentiation trajectories

The Monocle2 method utilizes unsupervised learning techniques on the transcriptome expression matrix of individual cells to categorize them into separate branches of the developmental path. Initially, we investigated the dynamics of CTHRC1 during cell differentiation, and subsequently employed Plot pseudotime heatmap to visualize gene expression patterns within the feature gene set across cellular evolution.

### Analysis of intercellular communication

The communication between cells is achieved through the interaction of ligands and receptors. Within the tumor microenvironment, intercellular communication plays a pivotal role in facilitating immune evasion by tumor cells. CellChat utilizes gene expression levels to infer the pathways and magnitude of intercellular communication, while also characterizing the interactions between ligands and receptors. Overexpressed genes were identified, screened for ligand-receptor pairs, and mapped onto the protein-protein interaction network. To ensure high-quality data, a filter was applied excluding communication relationships involving low-quality cells with a minimum cell count of 3.

### Multiple immunofluorescence assays

Based on bioinformatics discoveries, we corroborated our findings using multiple methodologies. Multiple immunofluorescence assays were utilized to authenticate the interactions between distinct molecules and cells.

### Statistical analysis

The associations between variables were assessed using Pearson or Spearman coefficients. T-tests were utilized to compare continuous variables with a normal distribution, whereas the Mann-Whitney U test was employed for variables that did not follow a normal distribution. Categorical variables were compared using either the chi-square test or Fisher exact test. All data analysis was performed using R software (version 4.1.2, available at https://www.r-project.org/). Statistical significance was determined based on a two-sided P-value threshold of less than 0.05 (*P < 0.05; **P < 0.01; ***P < 0.001; ****P < 0.0001).

## Result

### CTHRC1 showed unique features in TNBC

**Figure 1:**
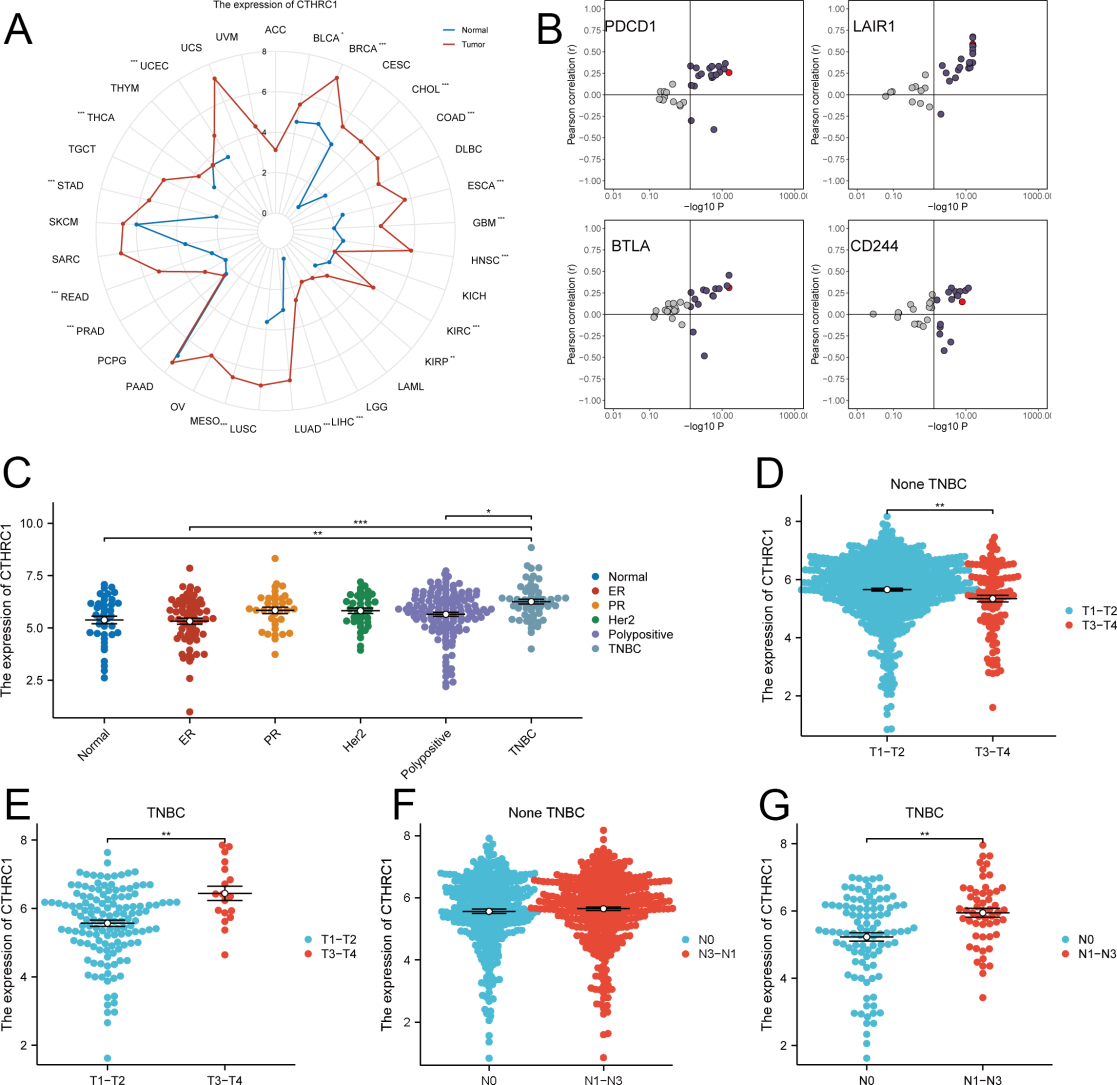
Expression analysis of CTHRC1 in multiple tumors. **(A)** CTHRC1 expression was evaluated in the majority of tumors. **(B)** Relationship between CTHRC1 and immune checkpoint inhibitory gene expression. **(C)** CTHRC1 expression was higher in TNBC than in other tissue types. **(D-G)** The expression of CTHRC1 was upregulated in triple-negative breast cancer with increasing tumor T-stage, N-stage, while no significant elevation was observed in non-triple-negative breast cancer. *P < 0.05; **P < 0.01; ***P < 0.001.

Significantly elevated expression levels of CTHRC1 were observed in breast, esophageal, and liver cancers compared to corresponding paraneoplastic tissues. Notably, breast cancer exhibited particularly high levels of CTHRC1 (**Figure 1A, B**). In non-TNBC tissues, the difference in CTHRC1 expression was not statistically significant. However, CTHRC1 expression was markedly elevated in TNBC tissues. Additionally, as the tumor T-stage and N-stage advanced, the expression of CTHRC1 exhibited an upward trend (**Figure 1C–G**). CTHRC1 was closely linked to the biology of TNBC.

### CTHRC1 was highly positively correlated with the invasion gene set

**Figure 2:**
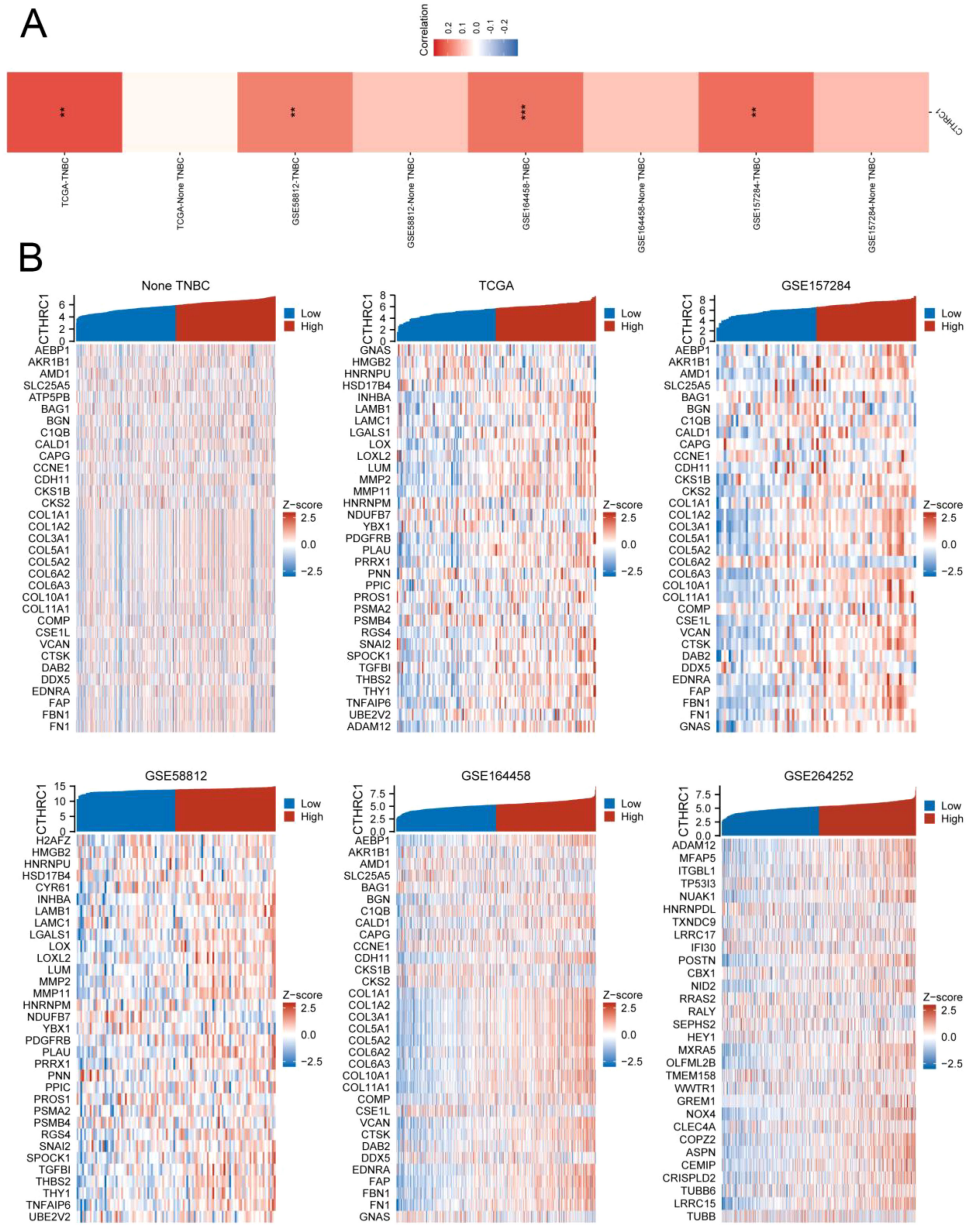
CTHRC1 showed a highly positive correlation with the tumour invasion gene set in TNBC. **(A)** CTHRC1 exhibited a significantly higher GSVA enrichment score in the tumour invasion gene set in TNBC tissues compared to non-TNBC tissues. **(B)** Invasive genes were upregulated concomitantly with increased CTHRC1 expression in triple-negative breast cancer, whereas no such correlation was observed in non-triple-negative breast cancer. **P < 0.01; ***P < 0.001.

For multiple samples, we utilized the GSVA enrichment score to evaluate the invasive gene set of tumors. The results demonstrated a strong positive correlation between CTHRC1 expression and tumor invasion scores specifically in TNBC. Conversely, no significant correlation was observed between CTHRC1 expression and tumor invasion scores in non-TNBC tissues. Conversely, there was no similar pattern found in non-TNBC. After conducting an in-depth analysis, we proceeded to investigate whether the invasive gene set displayed similar characteristics in both TNBC and non-TNBC. Intriguingly, the expression of a majority of genes within the invasive gene set exhibited an upward trend with elevated CTHRC1 gene expression specifically in the TNBC cohort (**Figure 2**). CTHRC1 was served as a uniquely distinctive biological molecule in TNBC relative to non-TNBC.

### CTHRC1 primarily affected macrophage infiltration

**Figure 3:**
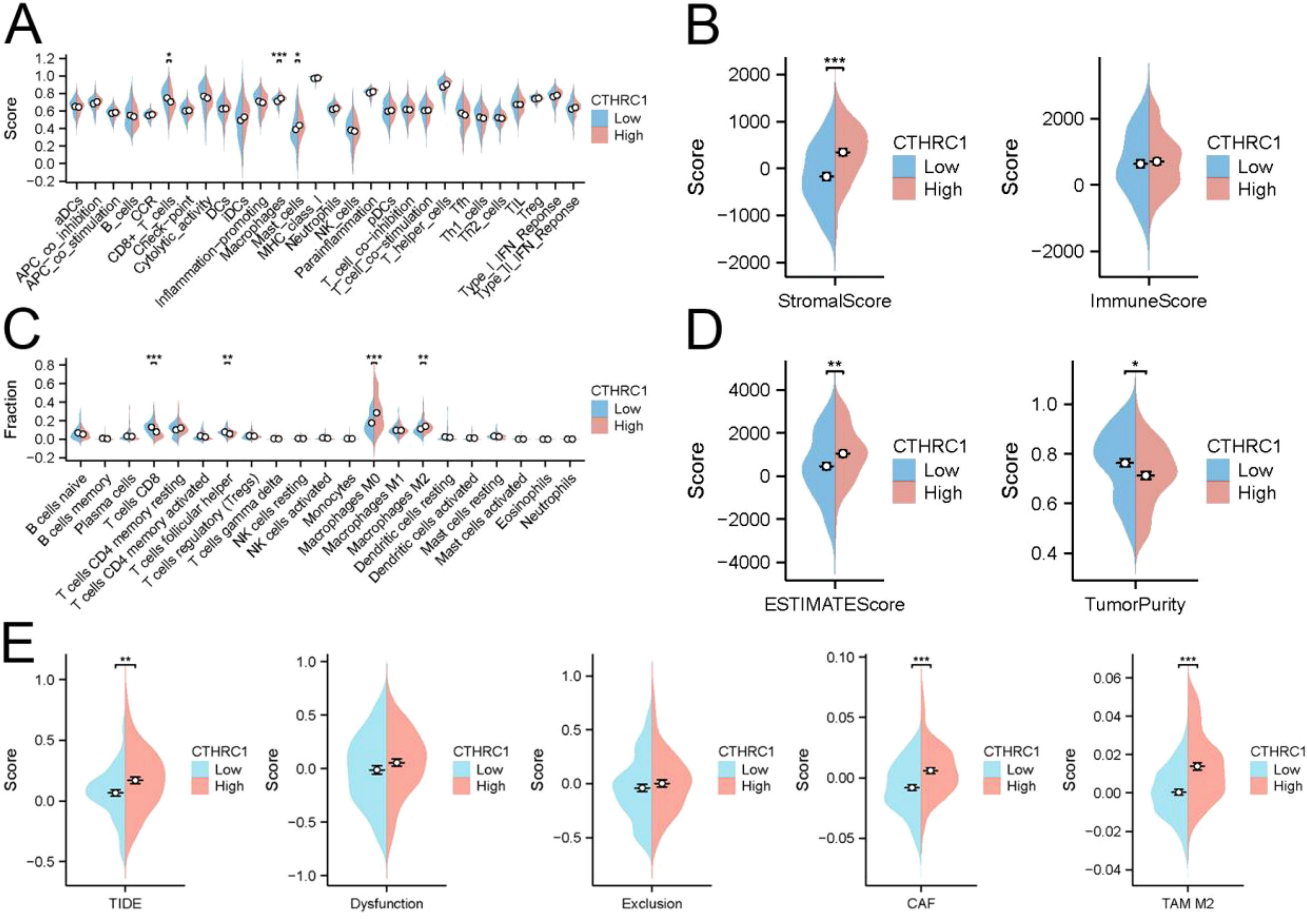
CTHRC1 regulated the immune microenvironment in triple-negative breast cancer by affecting M2-type macrophages. **(A)** The ssGSEA algorithm demonstrated the effect of CTHRC1 on immune cell function. **(B)** ESTIMATE of the algorithm to analyze the effect of CTHRC1 on triple-negative breast cancer. **(C)** The CIBERSORT algorithm demonstrated that increased CTHRC1 expression increased the abundance of M2-type macrophage infiltration. **(D)** ESTIMATE of the algorithm to analyze the effect of CTHRC1 on triple-negative breast cancer. **(E)** The TIDE algorithm showed that CTHRC1 affected patient immunotherapy efficacy. CTHRC1 primarily increased M2-type macrophages affecting the immune microenvironment, rather than by suppressing T-cell function. *P < 0.05; **P < 0.01; ***P < 0.001.

In TNBC, CTHRC1 exhibited limited association with most immune cells. However, there was a significant difference in the abundance of macrophage infiltration between the CTHRC1 high-expression and low-expression groups (**Figure 3A**). The infiltration abundance of M2-type macrophages significantly increased with higher levels of CTHRC1 expression (**Figure 3C**). The ESTIMATE algorithm indicated no disparities in immune scores but revealed discrepancies in mesenchymal scores between the high and low expressing groups of CTHRC1 (**Figure 3B, D**). TIDE predicted that patients in the high-expression group of CTHRC1 would derive less benefit from immunotherapy compared to those with lower expression levels. Similarly, while there were no variations observed in T-cell escape between the high and low expressing groups of CTHRC1, a difference was noted in M2-type macrophage scores (**Figure 3E**). CTHRC1 played a crucial role in the distinct tumour microenvironment of TNBC.

### CTHRC1 and invasion gene sets were primarily expressed in CAFs

The GSE176078 dataset comprises single-cell sequencing results from 9 patients diagnosed with TNBC. Each sample underwent rigorous quality control, and the data were integrated using the “Harmony” package in R programming language. Our study encompassed a total of 24272 cells and investigated expression patterns across 29626 genes. Cell type classification was performed based on established literature in the field of high-quality single-cell sequencing, revealing intriguingly predominant expression of both CTHRC1 and invasion-related genes in CAFs. Notably, no detectable expression was observed in immune cells, tumor-associated macrophages, or malignant cells. CTHRC1 exhibits a distinctive expression pattern in CAFs.

### CTHRC1 was associated with invasive gene set activity

**Figure 4:**
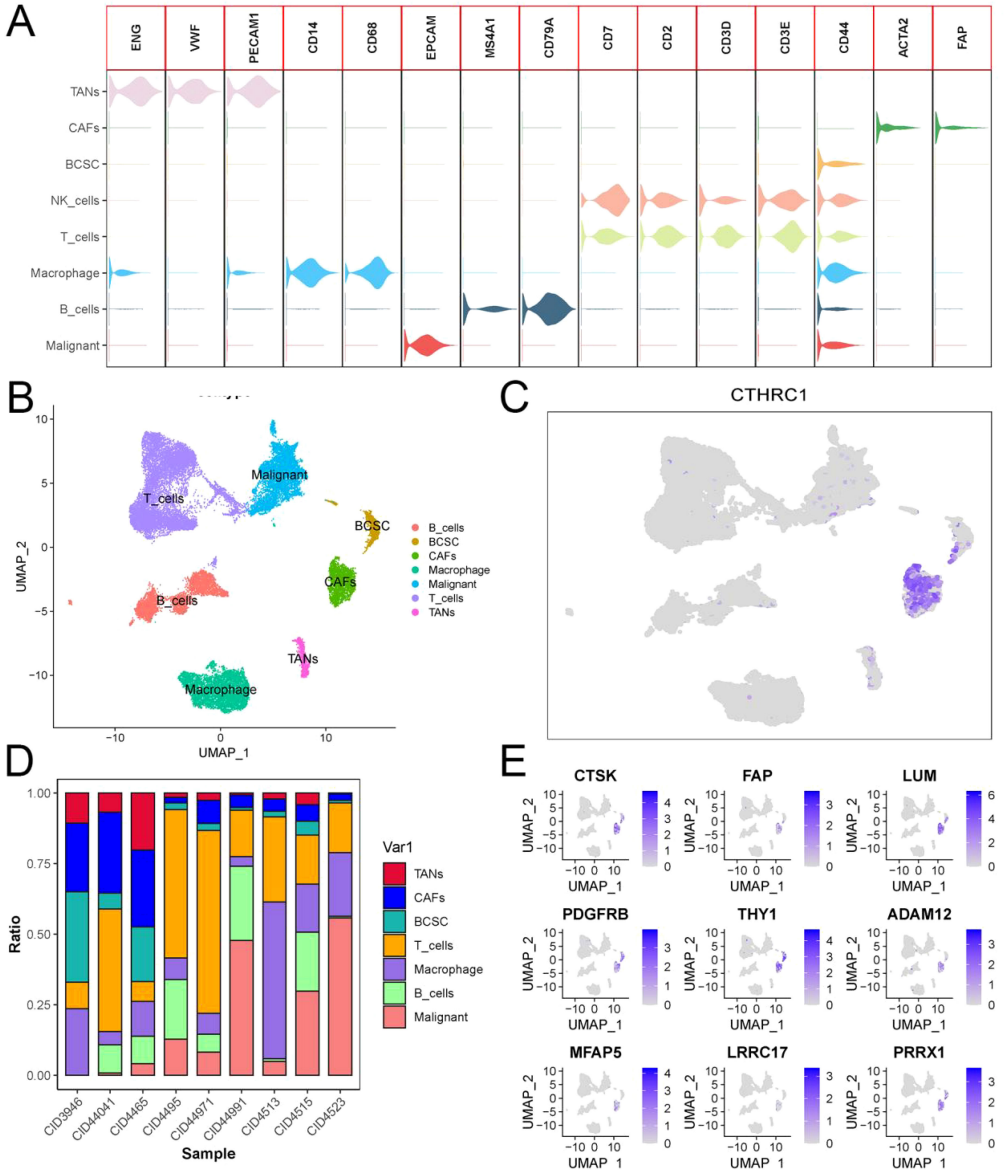
Analysis of single-cell sequencing data in triple-negative breast cancer. **(A, B)** Cluster cell annotation based on SingleR and previous literature. **(C)** CTHRC1 expression patterns in triple-negative breast cancer. **(D)** Plot of the proportion of cell types in single-cell sequencing samples. **(E)** Expression patterns of invasive genes in triple-negative breast cancer.

**Figure 5:**
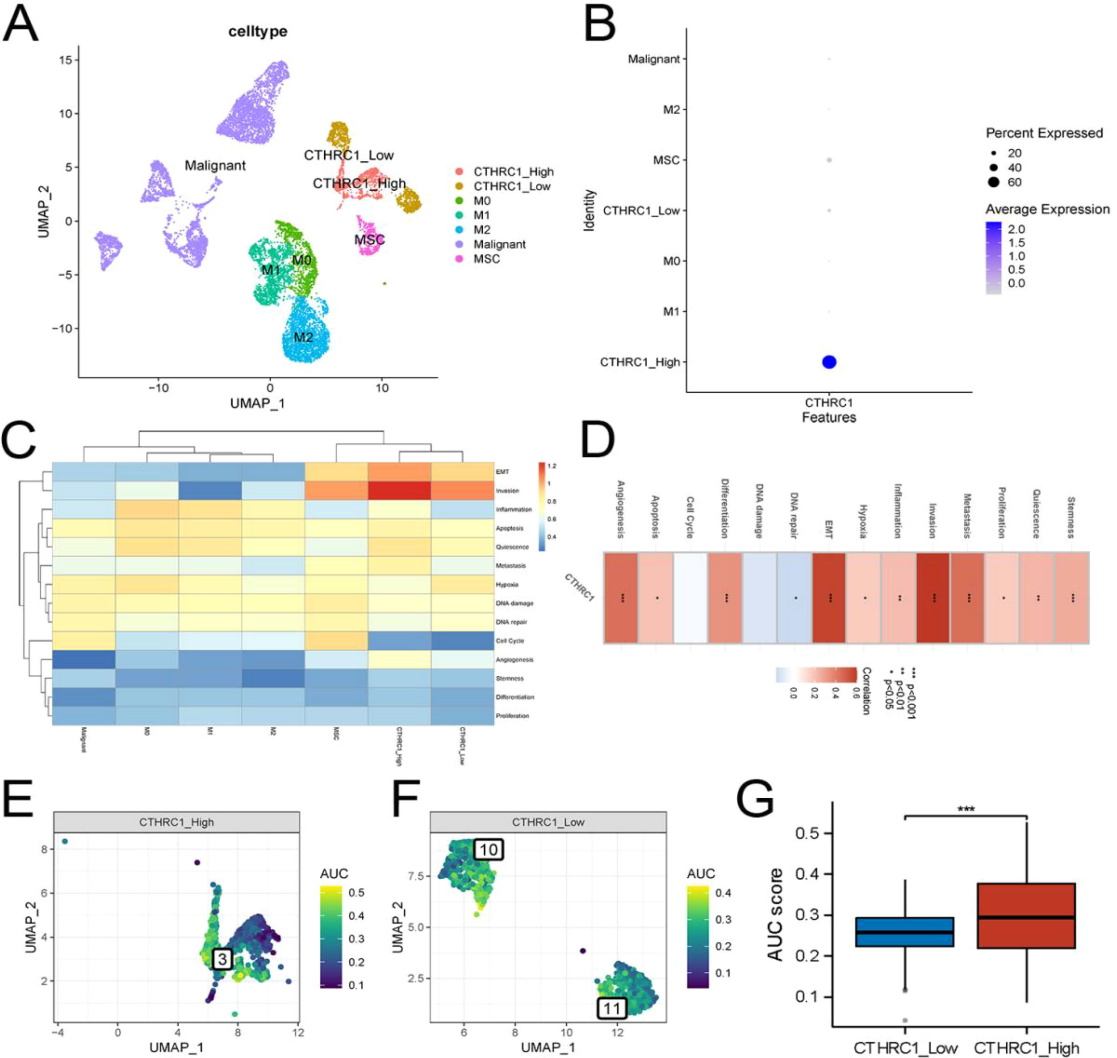
Invasive activity was strongest in CTHRC1-positive tumor-associated fibroblasts. **(A, B)** Re-clustering and annotation of tumor-associated fibroblasts and macrophages. **(C)** GSVA analysis of single-cell data demonstrated the strongest invasive activity of CTHRC1-positive fibroblasts. **(D)** Transcriptomic data revealed a strong positive correlation between CTHRC1 and invasive activity. **(E-G)** AUCell score exhibited higher invasive activity of CTHRC1-positive cells than CTHRC1-negative cells. *P < 0.05; **P < 0.01; ***P < 0.001.

Based on these findings, we isolated tumor-associated fibroblasts and macrophages from the single-cell data for recolonization analysis (**Figure 4A-E**). We identified three subtypes of macrophages, M0, M1 and M2. Furthermore, based on the expression levels of CTHRC1 in CAFs, they were categorized into high-expression and low-expression groups (**Figure 5A, B**). GSVA enrichment analysis of the single-cell data revealed that only cells in the high-expression group exhibited the highest activity of the invasive gene set (**Figure 5C, D**). To validate this observation, transcriptomic data was utilized to confirm a strong correlation between CTHRC1 expression and activity of genes associated with tumor invasion. Finally, AUCell was employed to score cellular invasion gene set activity, demonstrating a significantly higher score for the high expression group compared to the low expression group (**Figure 5E-G**). Single-cell analysis revealed a significant positive correlation between CTHRC1 expression and tumour invasion score.

### Invasion genes dynamically changed along with CTHRC1

**Figure 6:**
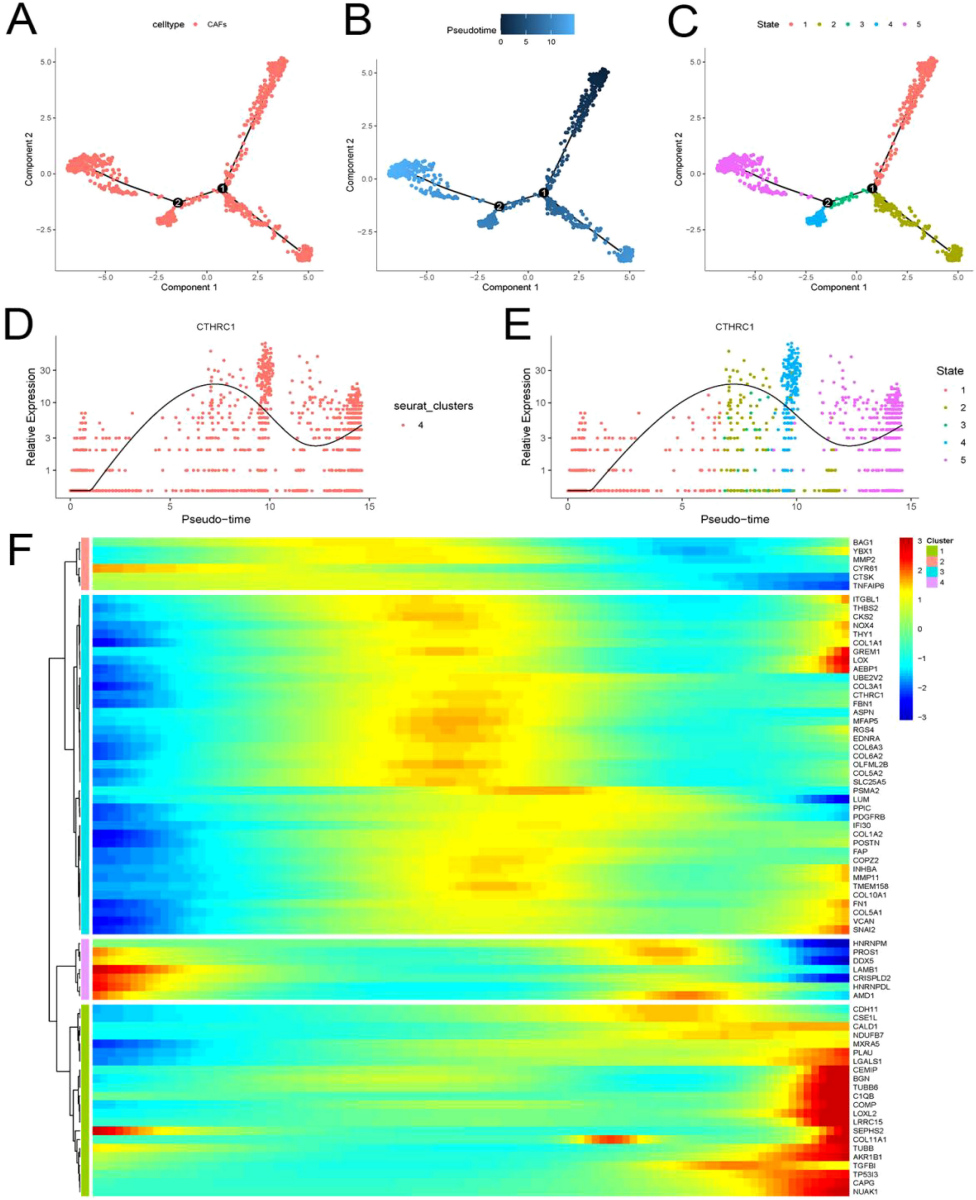
Invasive genes and CTHRC1 showed progressively higher expression with differentiation of tumor-associated fibroblasts. **(A-E)** CTHRC1 expression gradually increased with the differentiation of tumor-associated fibroblasts. **(F)** Invasive genes are accompanied by increased CTHRC1 expression in CAFs.

The tumor microenvironment undergoes dynamic changes. We utilized the “Monocle” package to elucidate the dynamic interplay between CTHRC1 and the invasive gene set (**Figure 6A-C**). CAFs were classified into five distinct differentiation trajectories, wherein the expression of CTHRC1 exhibited a significant upregulation concomitant with CAFs differentiation (**Figure 6D, E**). This augmented expression of CTHRC1 was accompanied by a notable increase in the expression levels of most members within the invasive gene sets (**Figure 6F**). Alterations in CTHRC1 expression were associated with modifications in the invasive activity of TNBC.

### CTHRC1 affected communication between CAFs and macrophages

**Figure 7:**
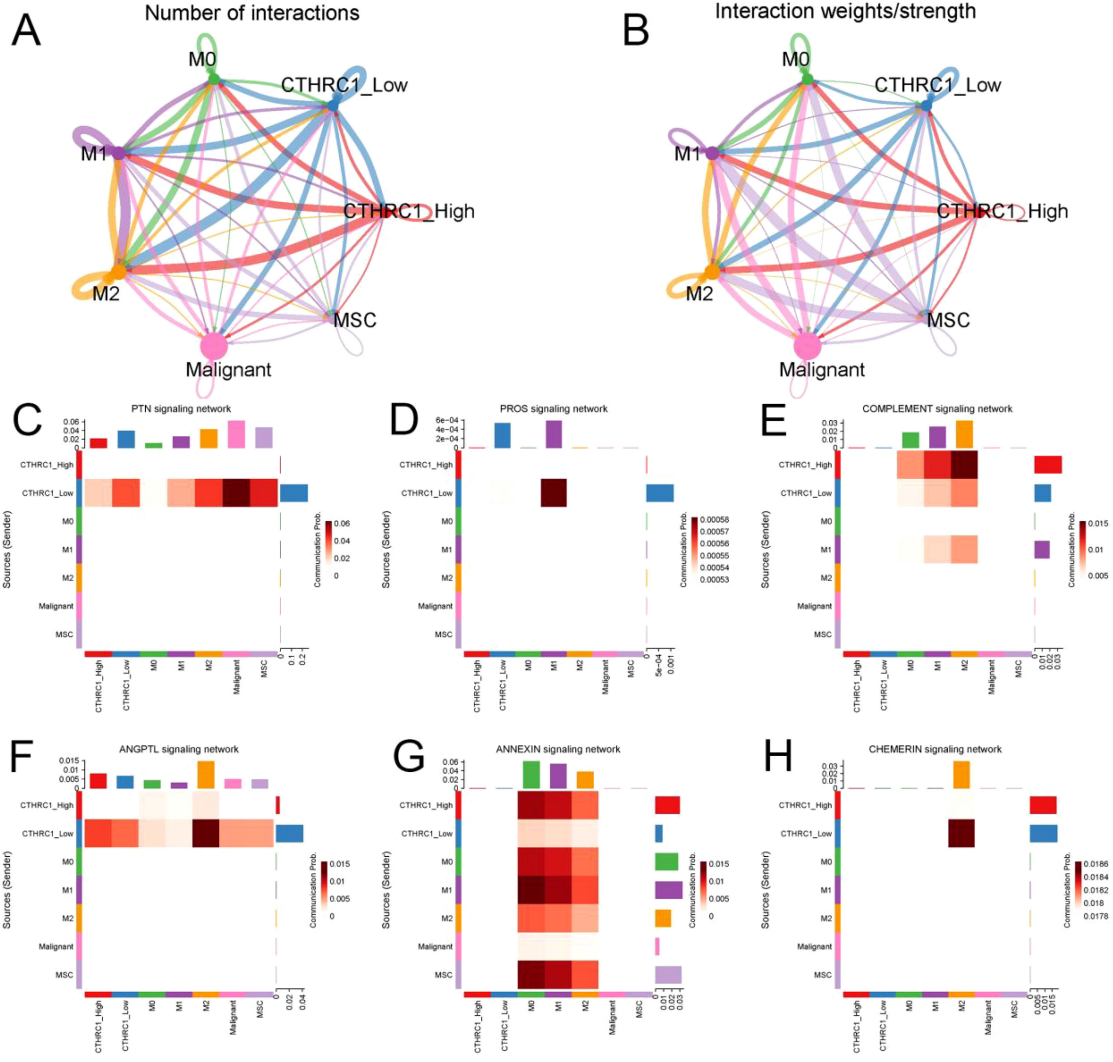
CTHRC1 expression level affected the interaction between tumor-associated fibroblasts and macrophages. **(A, B)** Probabilities and weights of interactions between tumor-associated fibroblasts and macrophages. **(C-H)** The high-expression group of CTHRC1 exhibited enhanced activity in the complement signaling pathway and annexin signaling pathway compared to the low-expression group of CTHRC1. The PTN signaling pathway, PROS signaling pathway, ANGPTL signaling pathway, and CHEMERIN signaling pathway showed high activity in CTHRC1 low group.

Receptor-ligand interactions serve as the foundation of intercellular communication. Utilizing the ‘Cellchat’ function for single-cell data analysis, potential signaling between cells was speculated upon (**Figures 7A, B**). CTHRC1 modulates communication between tumor-associated fibroblasts and macrophages. The group with high levels of CTHRC1 demonstrated increased functionality in the complement signaling pathway and annexin signaling pathway when compared to the group with low levels of CTHRC1. Conversely, a contrasting phenomenon was observed in the PTN signaling pathway, PROS signaling pathway, ANGPTL signaling pathway, and CHEMERIN signaling pathway (**Figure 7C-H**). CTHRC1-positive CFAs and macrophages exhibited close communication.

### CTHRCI-positive CAFs promoted infiltration of M2 macrophages

**Figure 8:**
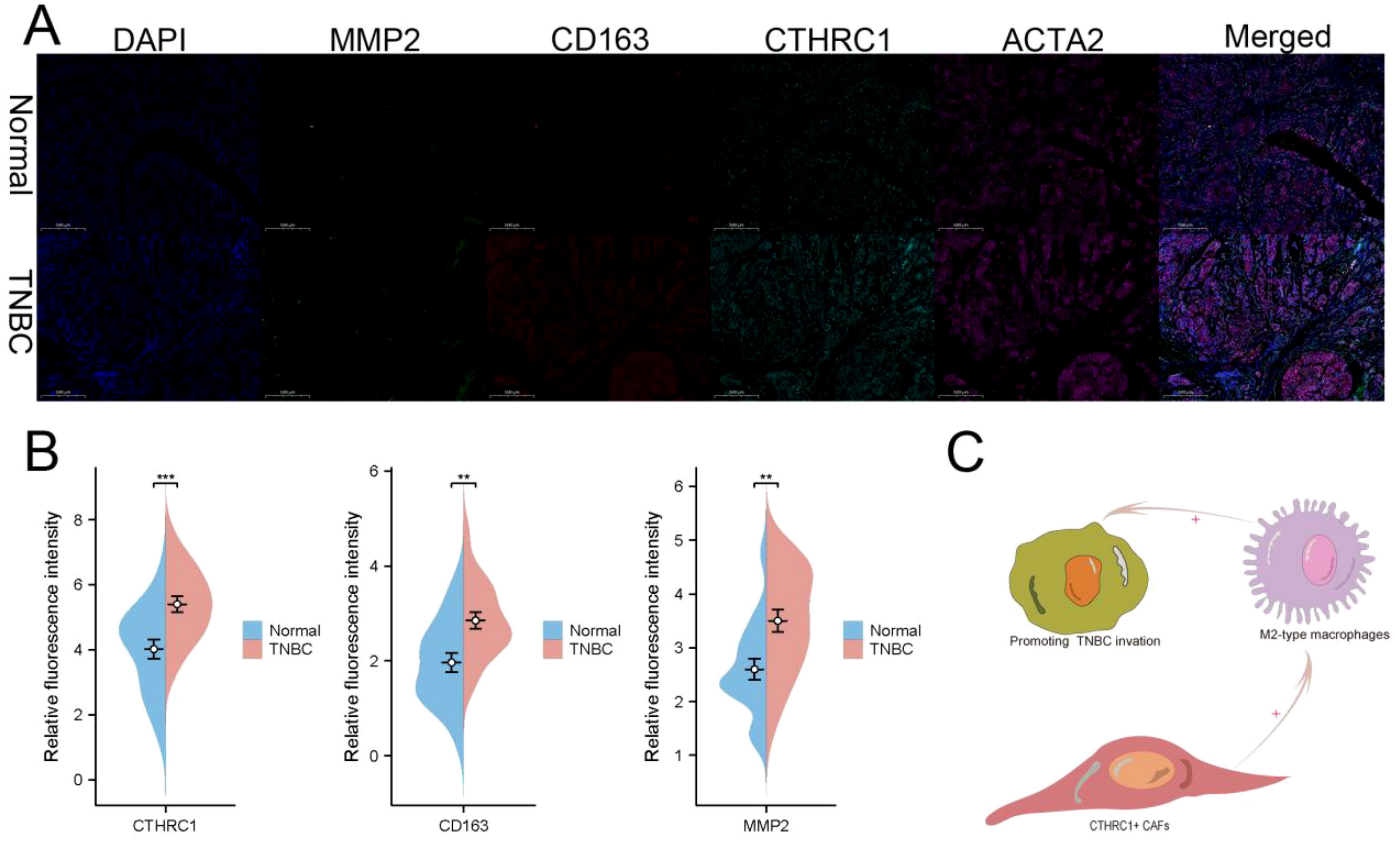
Immunofluorescence was used to validate the unique characteristics of CTHRC1 in TNBC patients. **(A)** Utilizing multiple immunofluorescence techniques, it was revealed that CTHRC1-positive fibroblasts exert an influence on the infiltration of M2-type macrophages. **(B)** CTHRC1, CD163, and MMP2 were upregulated in patients with TNBC. **(C)** A potential mechanism by which CTHRC1-positive CAFs regulated TNBC tumor microenvironment. **P < 0.01; ***P < 0.001.

The expression levels of CTHRC1 and invasive genes were increased in TNBC patients (**Figure 8B**). Immunofluorescence analysis of tumour and adjacent non-tumour tissues revealed a significant increase in M2 macrophage infiltration and elevated CTHRC1 expression levels (**Figure 8A**). These results validate our intriguing findings based on bioinformatics (**Figure 8C**). CTHRC1-positive CAFs facilitated the infiltration of M2-polarized macrophages.

## Discussion

TNBC accounts for approximately 20% of breast cancer patients (22). In contrast to other pathological types, triple-negative breast cancer exhibits distinctive clinical features and tumor microenvironmental characteristics (23). Statistical data indicates that the majority of patients experience recurrence within 5 years after diagnosis, implying that TNBC significantly worsen the burden on patients due to its high metastasis and recurrence rates (24). Considering the inter- and intra-tumor heterogeneity, identifying potential therapeutic targets based on the clinical characteristics of these tumors would greatly enhance patient prognosis (25, 26).

It is widely acknowledged that TNBC exhibits distinct invasive characteristics (27, 28). The investigation of potential targets to impede the invasion of TNBC is a highly researched topic (29, 30). SF3A2 and SNIP1 have been identified as crucial mediators implicated in the invasion of triple-negative breast cancer (31, 32). Diverse molecules and cellular components participate in regulating the microenvironment of TNBC and their malignant behaviors (33, 34). Ye et al. revealed that senescent CAFs facilitate the progression of TNBC by mediating immunosuppression (35). Mendez discovered that HMGA1 was predominantly enriched at the leading edges of primary tumor invasion and in metastatic lesions. Furthermore, inhibition of HMGA1 was found to attenuate invasion and metastasis in TNBC (36). Studies have demonstrated the involvement of CTHRC1 in gastric cancer metastasis and hepatocellular carcinoma metastasis (7, 37). Interestingly, CTHRC1-positive CAFs played a pivotal role in disease progression. Wong et al. observed that CTHRC1-expressing CAFs suppress immune cell function to promote prostate cancer progression (38).

We observed a strong association between CTHRC1 and tumor T-stage and N-stage specifically in TNBC, while no similar correlation was found in non-TNBC. The elevated expression of CTHRC1 in TNBC was accompanied by an increase in the expression of the invasive gene set, which was not observed in non-TNBC. Therefore, it was highly likely that CTHRC1 served as a signature molecule involved in invasion specifically in TNBC. Single-cell data analysis revealed predominant expression of CTHRC1 in CAFs, and GSVA enrichment analysis demonstrated that these fibroblasts exhibited the highest expression levels of the invasion gene set. Further clustering identified two distinct subpopulations within tumor-associated fibroblasts: CTHRC1-positive cells and negative cells. Interestingly, the activity of the invasive gene set was significantly higher in CTHRC1-positive CAFs compared to CTHRC1-negative CAFs. Notably, a positive correlation was observed between CTHRC1 expression and the differentiated expression of CAFs. Furthermore, during differentiation, there was a gradual increase in the expression of numerous CAFs accompanied by most of the invasive genes, providing further evidence for the role of CTHRC1 in promoting TNBC progression through regulation of CAFs.

The interactions between CTHRC1 and macrophages have been previously reported. Qin et al. demonstrated that CTHRC1 upregulated infiltration of M2-type macrophages through the TGF-β signaling pathway (39). Zhang et al. discovered that CTHRC1 regulated the abundance of M2-type macrophage infiltration, thereby reducing colon cancer liver metastasis (37). Similarly, our study observed intriguing phenomena where multiple immune infiltration analysis algorithms indicated a modest association between CTHRC1 and T cells, NK cells, and dendritic cells. However, multiple algorithms have demonstrated that CTHRC1 significantly enhances the abundance of M2-type macrophage infiltration. In addition, a comparative analysis was performed to examine the distinct interactions between macrophages and groups with high or low expression of CTHRC1. The results from single-cell intercellular communication analysis further validated that variations in CTHRC1 expression among CAFs can influence the reciprocal exchange with macrophages.

Although the concept behind this study is highly innovative and the results are remarkably intriguing, there are still certain limitations that need to be addressed. Firstly, while this study establishes a positive correlation between CTHRC1 and invasive gene set, it falls short in elucidating the underlying mechanism through which CTHRC1 regulates CAFs function. Secondly, the findings lack validation at both animal and cellular levels. Lastly, further investigation is warranted to comprehend the intricate interplay between CAFs and M2-type macrophages.

## Conclusion

In conclusion, CTHRC1 is predominantly expressed in CAFs and exhibits clinical characteristics resembling TNBC by influencing invasion-related genes. Moreover, CTHRC1 modulates the immunosuppressive tumor microenvironment of TNBC through the regulation of M2-type macrophages. However, further experimental validation is necessary to corroborate the findings of this study.

## Data Availability

The original contributions presented in the study are included in the article/**Supplementary Material**. Further inquiries can be directed to the corresponding author/s.
