# Supplementary figures and images for: Single-cell and transcriptome analyses revealed CTHRC1 a potential therapeutic target mediating invasion and tumor microenvironment in TNBC: experimental validation
Source: Front Immunol. 2025 Mar 11;16:1534981. doi: 10.3389/fimmu.2025.1534981 (PMC11933001; doi:10.3389/fimmu.2025.1534981)

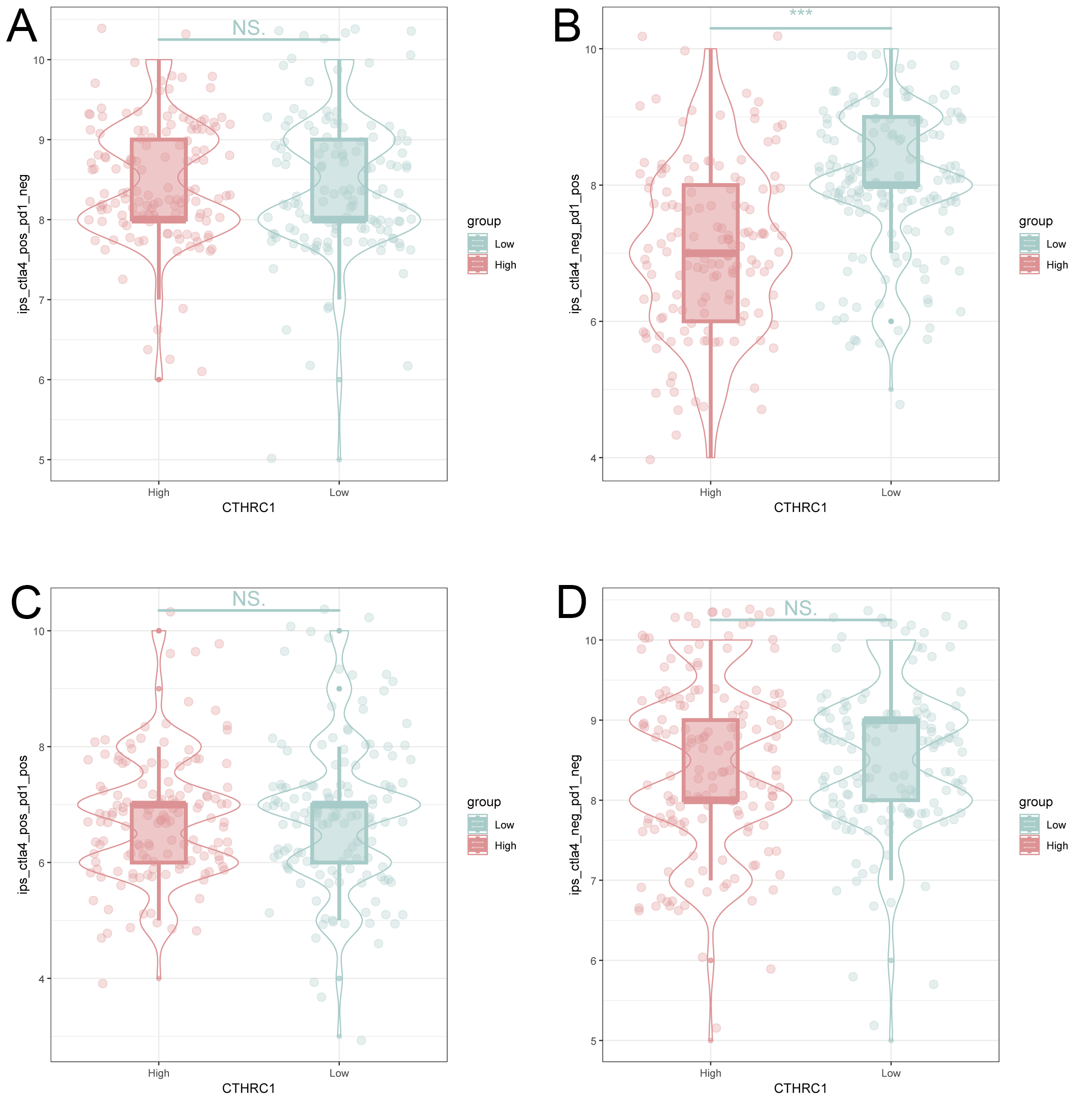

Supplement: Supplementary file 4 [file Image1.tif]
